# Exosomal lncRNA FAM225A accelerates esophageal squamous cell carcinoma progression and angiogenesis via sponging miR‐206 to upregulate NETO2 and FOXP1 expression

**DOI:** 10.1002/cam4.3463

**Published:** 2020-10-02

**Authors:** Chunyu Zhang, Yan Luo, Jingjing Cao, Xiaoyu Wang, Zhiwei Miao, Guoqing Shao

**Affiliations:** ^1^ Zhangjiagang TCM Hospital Affiliated to Nanjing University of Chinese Medicine Zhangjiagang P.R. China

**Keywords:** angiogenesis, esophageal squamous cell carcinoma, exosomal, FAM225A

## Abstract

Esophageal cancer is one of the leading causes of cancer‐related deaths worldwide. FAM225A is a novel lncRNA, only has been explored in nasopharyngeal carcinoma tumorigenesis. This study aims to investigate the regulatory mechanism of FAM225A in esophageal squamous cell carcinoma (ESCC). We discovered that FAM225A exhibited higher expression in ESCC. The silence of FAM225A attenuated cell viability, migration, and invasion, but facilitated cell apoptosis in ESCC. Exosome‐mediated transfer of lncRNA FAM225A could participate in ESCC progression. In addition, we found that miR‐206 bound to FAM225A. Moreover, we further demonstrated that FAM225A absorbed miR‐206 to upregulate NETO2 and FOXP1 expression, and FOXP1 acted as a transcription factor to enhance FAM225A expression. Eventually, it was revealed that the overexpression of NETO2 or FOXP1 rescued the effects of FAM225A repression on ESCC progression. Our results suggested that FAM225A upregulated NETO2 and FOXP1 expression by sponging miR‐206 to accelerate ESCC progression and angiogenesis. These results determined the biological role of lncRNA FAM225A in ESCC tumorigenesis, and FAM225A may be a promising biomarker for ESCC treatment.

## Graphical abstract

FAM225A up‐regulated NETO2 and FOXP1 expression by absorbing miR‐206 in ESCC. FOXP1 induced FAM225A expression to promote ESCC progression.

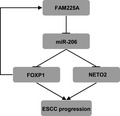

## 1 INTRODUCTION

Esophageal cancer (EC) represents one of the most common lethal malignancies.^1^, ^2^, ^3^ The two subtypes of EC are esophageal adenocarcinoma (EAC) and Esophageal squamous cell carcinoma (ESCC). ESCC accounts for 90% of all EC cases and is the primary type of EC.^4^ At present, a lot of molecular regulatory mechanisms underlying ESCC progression remain unclear. Therefore, it is urgent to look for better biomarkers for ESCC treatment.

Long non‐coding RNAs (lncRNAs) are deemed as a group of mature RNAs with longer than 200 nts, which have no protein‐coding ability.^5^, ^6^ LncRNAs have been illustrated to be involved in the pathogenesis of most cancers. For example, lncRNA MEG3 suppresses chronic myeloid leukemia cell proliferation.^7^ Upregulated SNHG1 facilitates osteosarcoma progression via activating Wnt/β‐catenin pathway.^8^ LncRNA‐p23154 regulates Glut1‐mediated glycolysis to accelerate oral squamous cell carcinoma cell invasion.^9^ FAM225A, a novel lncRNA, has only been proved to accelerate the tumorigenesis and metastasis in nasopharyngeal carcinoma by serving as a ceRNA to interact with miR‐590‐3p/miR‐1275 and increasing TGB3 expression.^10^ Nevertheless, the FAM225A‐mediated regulatory mechanism in ESCC remains obscure.

A great number of researches have demonstrated that microRNAs (miRNAs) can be regulated by lncRNAs and directly bind to their target mRNAs, thus causing translational suppression or mRNA degradation.^11^ Abundant evidence uncovered that miRNAs were implicated in the tumorigenesis of human cancers, including ESCC. For instance, miR‐296‐5p represses ESCC progression via targeting STAT3.^12^ MiR‐92a‐3p regulates PTEN to enhance cell viability, migration, and invasion in ESCC.^13^ MiR‐874‐3p acts as a prognostic factor and targets STAT3 to exhibit its tumor suppressor in ESCC.^14^ MiR‐206 has been revealed to function as a tumor suppressor in various tumors. MiR‐206 restrains cell growth and invasion in ovarian cancer by retarding the c‐Met/AKT/mTOR pathway.^15^ MiR‐206 weakened breast cancer cell stemness and metastasis via regulating the MKL1/IL11 pathway.^16^ MiR‐206 modulates CXCL11 to suppress prostate cancer cell proliferation and migration.^17^ Nevertheless, there is no research on the role of miR‐206 in ESCC.

In our study, we validated that FAM225A enhanced NETO2 and FOXP1 expression by absorbing miR‐206 in ESCC. This finding may offer a novel perspective for the pathological mechanism of ESCC.

## 2 MATERIALS AND METHODS

### 2.1 Clinical samples

Thirty pairs of ESCC tissues and paracancerous tissues were acquired from patients in Zhangjiagang TCM Hospital Affiliated to the Nanjing University of Chinese Medicine. Written consents from all participators were acquired before this work. All tissues were instantly refrigerated in liquid nitrogen and then maintained at −80℃. Our work was approved by the ethics committee of Zhangjiagang TCM Hospital Affiliated to the Nanjing University of Chinese Medicine.

### 2.2 Cell culture

Human ESCC cell lines (ECA109, TE‐1, KYSE150, and KYSE‐410), human esophageal epithelial cell line (HET‐1A), and human umbilical vein endothelial cells (HUVECs) were obtained from the American Type Culture Collection (ATCC, USA). DMEM (Gibco; Thermo Fisher Scientific, Inc) added with penicillin (100  U/mL; Beyotime), streptomycin (100 μg/mL; Beyotime) plus 10% FBS (PAN‐Biotech) was used to incubate above cells. HUVECs were cultured in a humidified incubator containing 5% CO_2_ at 37℃.

### 2.3 Cell transfection

Short hairpin RNAs (shRNAs) targeting FAM225A (sh‐FAM225A#1; 5′‐ GCAGCUCCAUUUCUGGUGUUU‐3′ and sh‐FAM225A#2; 5′‐ CCCAGAUCAUCUUUGACACAU‐3′) or negative control (sh‐NC; 5′‐ AAUUCUCCGAACGUGUCACGU‐3′) was transfected into ECA109 and TE‐1 cells. MiR‐206 mimics (5ʹ‐UGGAAUGUAAGGAAGUGUGUGG‐3ʹ) and miR‐NC (5ʹ‐UUCUCCGAACGUGUCACGUUU‐3ʹ) were synthesized from Genechem (Shanghai, China). The pcDNA3.1, pcDNA3.1‐FOXP1, and pcDNA3.1‐NETO2 plasmids were all obtained from GeneChem. The transfection was performed with Lipofectamine 2000 (Invitrogen).

### 2.4 RT‐QPCR

Total RNAs were isolated from ESCC cells by TRIzol reagent ((Invitrogen). The reverse transcriptase kit (Takara) or the TaqMan^®^ miRNA reverse transcription kit (Thermo Fisher Scientific) was applied to reverse‐transcribe the RNAs to cDNAs. RT‐qPCR was performed using the SYBR‐Green PCR Master Mix kit (Takara, Dalian, China). The relative expression of genes was calculated with the use of the 2^−ΔΔCt^ method. GAPDH and U6 served as internal references. The following primers were used: FAM225A: forward, 5ʹ‐TTAGTTGATGCCAAAGAGTTCC‐3ʹ, and reverse, 5ʹ‐ATCCATACTCCAAGTAACAGAC‐3ʹ; miR‐206: forward, 5ʹ‐ TGTAAACATCCTACACTCTCAGCAA‐3ʹ, and reverse, 5ʹ‐ GCTGTCAACGATACGCTACGTAACG‐3ʹ; FOXP1: forward, 5ʹ‐ TCCAGAAACATCTAAGCTGGA‐3ʹ, and reverse, 5ʹ‐GGAGGATCACTTGAAGCCA‐3ʹ; NETO2: forward, 5ʹ‐TCTTGTGGTATGGAACTATGTG‐3ʹ, and reverse, 5ʹ‐ATCCGCATCAAAGTGTGAG‐3ʹ; GAPDH: forward, 5ʹ‐ CTGGGCTACACTGAGCACC‐3ʹ, and reverse, 5ʹ‐ AAGTGGTCGTTGAGGGCAATG‐3ʹ; U6: forward, 5ʹ‐CCGCCCGCCGCCAGGCCCC‐3ʹ, and reverse, 5ʹ‐ ATATGGAACGCTTCACGAATT‐3ʹ.

### 2.5 Exosomes isolation

An ExoQuick Exosome Precipitation Solution kit (SBI, System Biosciences, CA) was applied to separate exosomes from the ESCC cell culture medium. Finally, the pellets contained with exosomes were collected.

### 2.6 CCK‐8

ECA109 and TE‐1 cells (1 × 10^3^ cells/well) were plated on a 96‐well plate. After being incubated for 0, 24, 48, and 72 hours, CCK‐8 solution (10 μL, Dojindo) was mixed in each well. The absorbance at 450 nm was examined through a microplate reader (BioTek).

### 2.7 Tunel

TUNEL Apoptosis Kit (Roche, Mannheim, Germany) was used to analyze cell apoptosis. After dehydrating by ethanol, TUNEL reaction mixture (Roche) was utilized to dye and incubate ECA109 and TE‐1 cells. Nuclear staining was performed using DAPI. TUNEL‐positive cells were observed through a microscope (Olympus).

### 2.8 Wound healing assay

ESCC cells were plated in 6‐well plates and indicated to reach 70% confluence for the wound healing assay. A 200 μL pipette tip was applied to generate artificial scratches. Images of migrated cells were observed at 0 and 24 hours with the application of a microscope (Leica DMI4000B, Milton Keynes).

### 2.9 Transwell

ESCC cell invasion was measured with the use of Transwell chambers (8.0 μm pore size; EMD Millipore) and Matrigel (Corning Inc). Briefly, free‐medium contained cells were added into the upper chamber coated with Matrigel, and 600 uL DMEM medium contained 10% FBS was added into the bottom chamber. After 48 hours, cells in the lower membrane were dyed with 0.1% crystal violet. Invaded cells were counted under a microscope (ZEISS).

### 2.10 Tube formation assay

About 50 μL of Matrigel (BD Biosciences) was added to each well of 96‐well plate. HUVECs were polymerized for 30 minutes at 37℃, and next harvested from the co‐culture system. Matrigel‐prepared 96‐well plate was added with HUVECs (1 × 10^4^ cells per well). After incubating for 4 hours, an IX71 inverted microscope was used to visualize the tubes.

### 2.11 Luciferase reporter assay

The pmirGLO‐FAM225A‐WT/Mut, pmirGLO‐NETO2‐WT/Mut, and pmirGLO‐FOXP1‐WT/Mut reporters were produced from GenePharma (Shanghai, China). MiR‐206 mimics or miR‐NC was co‐transfected with these above reporters into ESCC cells. The pGL3‐FAM225A‐Pro‐WT/Mut reporters were co‐transfected with pcDNA3.1‐FOXP1 or pcDNA3.1 vectors into ESCC cells. Upon co‐transfection for 48 hours, the relative luciferase activity was acquired via a dual‐luciferase reporter assay system (Promega).

### 2.12 Western blot

ESCC cells were lysed in RIPA buffer to isolate total proteins. Next, these proteins were separated by SDS‐PAGE (Millipore, Bedford, MA, USA), then moved to PVDF membranes. After blockage with milk, membranes were incubated with the primary antibodies, such as anti‐PCNA, anti‐CyclinD1, anti‐Bax, anti‐Bcl‐2, anti‐MMP‐2, anti‐MMP‐9, anti‐E‐cadherin, anti‐Vimentin, anti‐CD63, anti‐CD81, AND anti‐GAPDH. ECL chemiluminescent detection system was applied to make immunoblots to be visualized.

### 2.13 Statistical analysis

Data were presented as the mean ± standard deviation (SD) from three repeated experiments and analyzed using SPSS 19.0 software (SPSS Inc, USA) and GraphPad Prism (GraphPad Software, USA). Differences between groups were assessed by Student's *t* test or one‐way ANOVA, followed by Tukey's post hoc test. Spearman's correlation analysis was used for analyzing the correlations between genes. The overall survival was assessed by the Kaplan–Meier method. *P* < .05 was deemed statistically significant.

## 3 RESULTS

### 3.1 FAM225A exhibited higher expression in ESCC and facilitated ESCC progression

**FIGURE 1:**
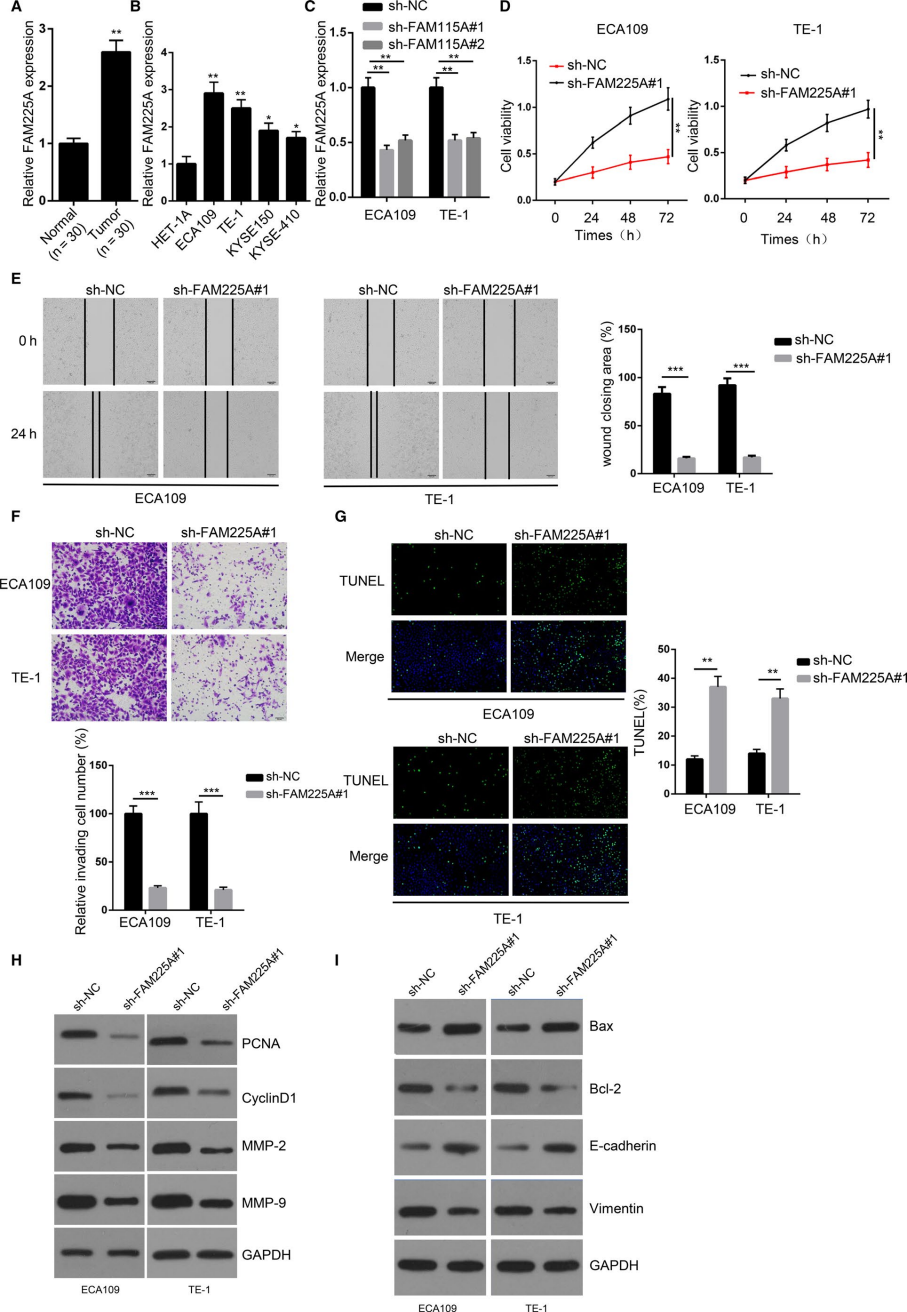
FAM225A exhibited higher expression in ESCC tissues and cell lines to facilitate ESCC progression. A‐B, RT‐qPCR assay was applied to detect the expression of FAM225A in ESCC tissues (n = 30) and cell lines. C, RT‐qPCR assay was applied to detect the transfection efficiency of sh‐NC, sh‐FAM225A#1, and sh‐FAM225A#2 in ECA109 and TE‐1 cells. D, CCK8 assay was used to measure cell proliferation in ECA109 and TE‐1 cells after knocking down FAM225A. E‐F, Wound healing and Transwell assays were performed to assess cell migration and invasion in ECA109 and TE‐1 cells after suppressing FAM225A. G, TUNEL assay was conducted to verify cell apoptosis in ECA109 and TE‐1 cells after FAM225A silence. H‐I, Western blot assay was devoted to test the expression of cell proliferation‐related proteins (PCNA and CyclinD1), cell apoptosis‐related proteins (Bax and Bcl‐2), and metastasis‐related proteins (MMP‐2, MMP‐9, E‐cadherin and Vimentin) in ECA109 and TE‐1 cells after inhibiting FAM225A. GAPDH serves as the loading control. *P < .05, **P < .01, ***P < .001

To verify the biological function of FAM225A in ESCC, FAM225A expression was first assessed by RT‐qPCR analysis. It was revealed that FAM225A was remarkably overexpressed in ESCC tissues (n = 30) (Figure 1A). Moreover, the high expression of FAM225A was markedly correlated with tumor size, TNM stage, and lymph node status (Table 1). It was also validated that FAM225A expression was upregulated in ESCC cell lines (ECA109, TE‐1, KYSE150, and KYSE‐410) (Figure 1B). The knockdown efficiency of sh‐FAM225A#1 or sh‐FAM225A#2 is shown in Figure 1C. Cell proliferation was reduced after suppressing FAM225A (Figure 1D). In addition, cell migration and invasion were both attenuated by FAM225A knockdown (Figure 1E‐F). In the TUNEL assay, we discovered that cell apoptosis was enhanced in sh‐FAM225A#1 group (Figure 1G). Besides, we assessed the expression levels of cell viability‐related proteins (PCNA and CyclinD1), cell apoptosis‐related proteins (Bax and Bcl‐2), and metastasis‐related proteins (MMP‐2, MMP‐9, E‐cadherin, and Vimentin). It was found that Bax and E‐cadherin expression was increased, but Bcl‐2, PCNA, CyclinD1, MMP‐2, MMP‐9, and Vimentin expression was decreased by knocking down FAM225A (Figure 1H‐I). In sum, the above data indicated that FAM225A exhibited higher expression in ESCC tissues and cell lines, and facilitated ESCC progression by accelerating cell viability, migration, and invasion as well as repressing cell apoptosis.

**TABLE 1:** The correlation between FAM225A expression and clinicopathologic features of ESCC patients

Clinicopathological features		n	lncRNA FAM225A		*P*‐value
			High(n = 14)	Low(n = 16)	
Gender	Male	17	8	9	.331
	Female	13	6	7	
Age (y)	＜60	11	5	6	.748
	≥60	19	9	10	
Tumor size	≤2 cm	12	8	4	.031
	＞2 cm	18	6	12	
TNM stage	<III stage	10	5	4	＜.001
	≥III stage	20	9	12	
Lymph node status	Yes	18	6	12	＜.001
	No	12	8	4	

### 3.2 Extracellular FAM225A was packaged into exosomes in ESCC cells

**FIGURE 2:**
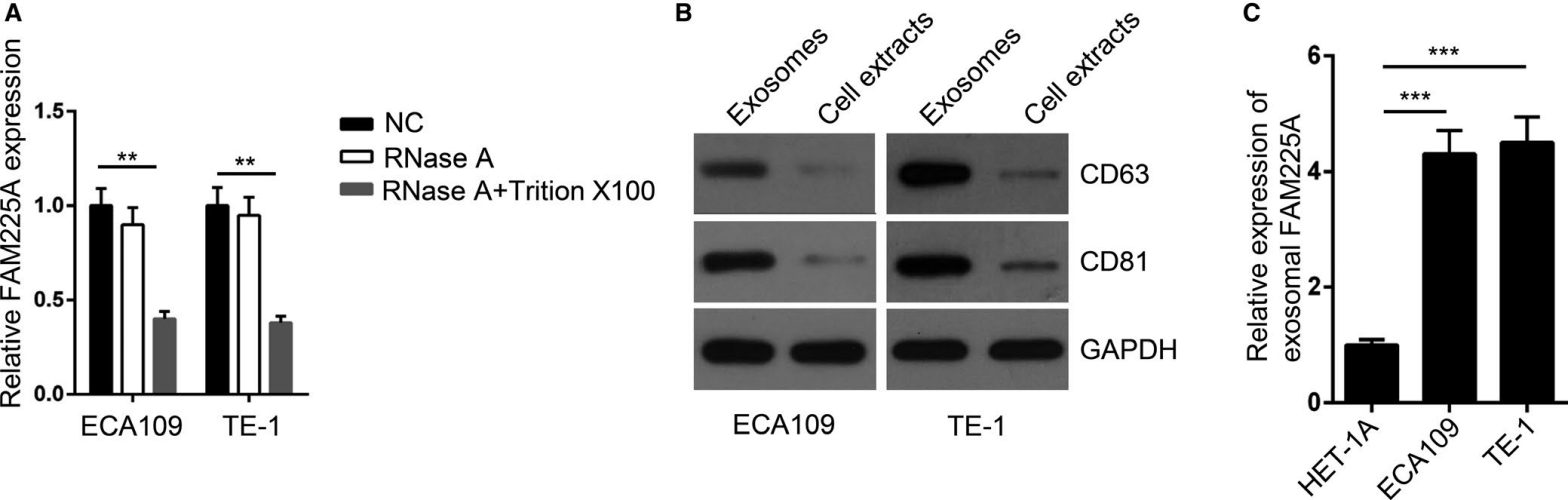
Exosome‐mediated transfer of lncRNA FAM225A accelerated angiogenesis. A, RT‐qPCR assay was performed to measure the expression of FAM225A in control, RNase A or RNase A + Triton X‐100 group. B, Western blot assay was used to detect the expression of exosomal markers (CD63 and CD81) in purified exosomes and exosome‐depleted cell extracts. GAPDH serves as a loading control. C, RT‐qPCR analysis was applied to detect FAM225A expression of cell culture supernatant exosomes from ECA109 and TE‐1 cells. **P < .01, ***P < .001

A large variety of cell types could secrete exosomes and culture medium might include lncRNAs‐contained exosome. To explore whether FAM225A was secreted by packaging into exosomes, the FAM225A expression was measured after treating with RNase A or Triton X‐100. The results demonstrated that RNase A treatment had no effect on the expression of FAM225A, while RNase A + Triton X‐100 treatment significantly decreased FAM225A expression in the culture medium (Figure 2A). This discovery suggested that lncRNA FAM225A was not directly secreted but protected by the membrane. The exosomal markers (CD63 and CD81) were assessed by western blot, indicating that exosomes were successfully enriched (Figure 2B). The expression of FAM225A was higher in supernatant exosomes in the culture medium of ESCC cell lines (Figure 2C).

### 3.3 FAM225A absorbed MIR‐206 in ESCC

**FIGURE 3:**
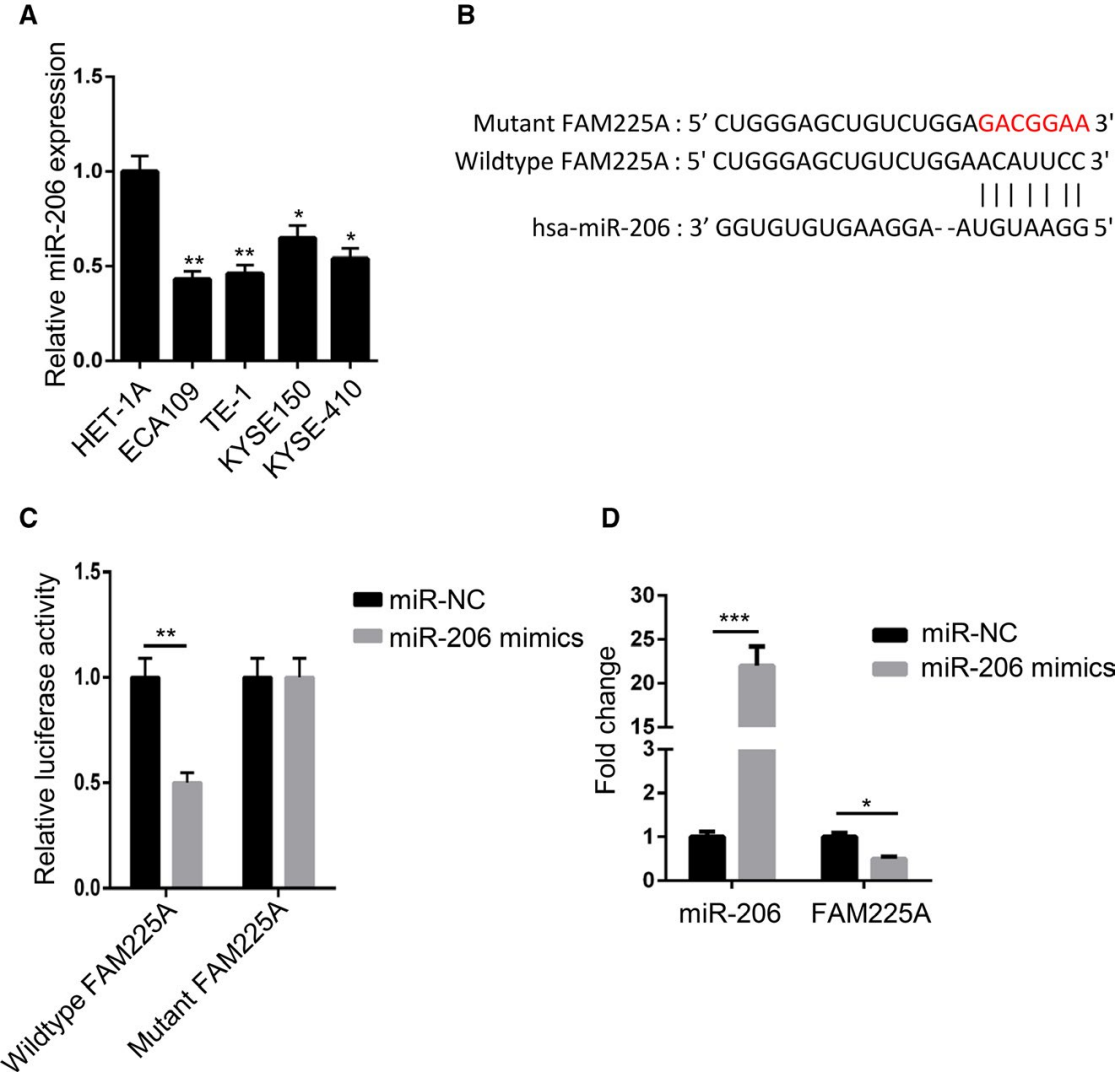
FAM225A absorbed miR‐206 in ESCC. A, RT‐qPCR assay was applied to assess the expression of miR‐206 in ESCC cell lines. B, Binding sequences between FAM225A and miR‐206 were predicted by the starBase bioinformatic analysis website. C, Luciferase reporter assay was adopted to verify the binding ability between FAM225A and miR‐206 in ECA109 cells. D, RT‐qPCR assay was performed to detect the expression of miR‐206 and FAM225A in ECA109 cells after overexpressing miR‐206. *P < .05, **P < .01, ***P < .001

RT‐qPCR results illustrated that miR‐206 expression was apparently reduced in ESCC cell lines (Figure 3A). Using the starBase bioinformatic analysis website, FAM225A was suggested to bind with miR‐206 via complementary base pairing (Figure 3B). To verify whether FAM225A could bind with miR‐206, we conducted a luciferase gene reporter assay. The findings indicated that miR‐206 mimics weakened the luciferase activities of pmirGLO‐FAM225A‐WT vectors but had no effect on the luciferase activities of pmirGLO‐FAM225A‐Mut vectors (Figure 3C). Moreover, the upregulation of miR‐206 reduced FAM225A expression (Figure 3D). These findings revealed that FAM225A absorbed miR‐206 in ESCC.

### 3.4 NETO2 and FOXP1 were downstream target genes of MIR‐206

**FIGURE 4:**
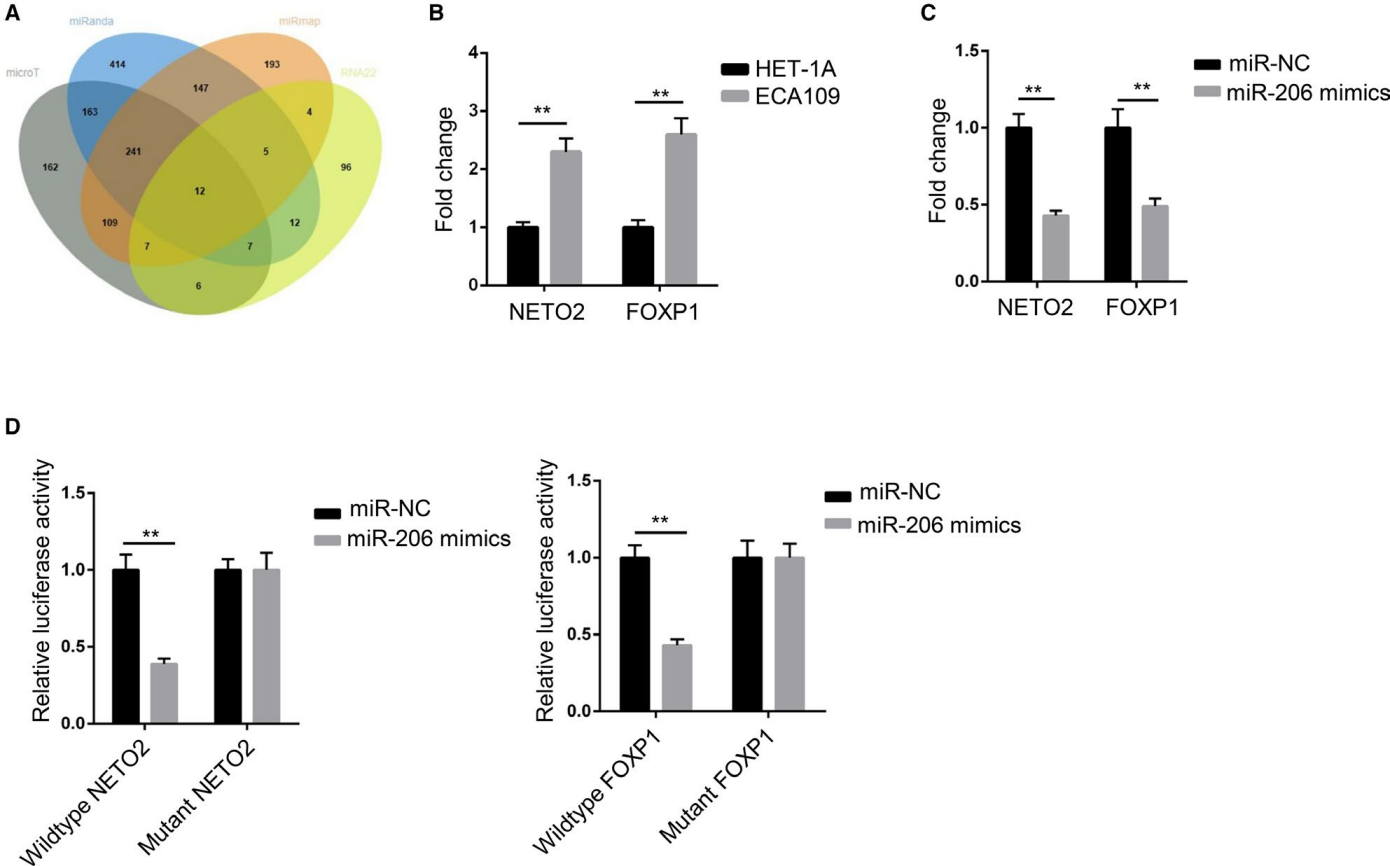
NETO2 and FOXP1 were downstream target genes of miR‐206. A, Venn diagram was used to display mRNAs which could bind with miR‐206. B, RT‐qPCR assay was applied to assess the expression of NETO2 and FOXP1 in HET‐1A and ECA109 cell lines. C, RT‐qPCR assay was performed to measure the expression of NETO2 and FOXP1 in ECA109 cells after upregulating miR‐206. D, Luciferase reporter assay was devoted to verify interactions between miR‐206 and NETO2 (or FOXP1) in ECA109 cells. ^**^ *P* < .01

As shown in the Venn diagram, 12 potential target genes (OAS2, SPRED1, NETO2, ATXN1L, DDX5, SMAD4, DDX18, MAPK1, FOXP1, PTPRG, TBC1D9, and PGM2) that could bind with miR‐206 were displayed in the overlap region (Figure 4A). We examined 12 mRNAs expression in ECA109 and HET‐1A cells. RT‐qPCR results showed that the NETO2 and FOXP1 expression were markedly higher in ECA109 cells than that in HET‐1A cells (Figure 4B). After overexpressing miR‐206, NETO2 and FOXP1 levels were markedly decreased (Figure 4C). Moreover, miR‐206 mimics undermined the luciferase activities of pmirGLO‐NETO2‐WT and pmirGLO‐FOXP1‐WT vectors, but there was no effect on the luciferase activities of pmirGLO‐NETO2‐Mut and pmirGLO‐FOXP1‐Mut vectors (Figure 4D). Taken together, NETO2 and FOXP1 were downstream target genes of miR‐206 in ESCC.

### 3.5 FOXP1 induced FAM225A expression to promote ESCC progression

**FIGURE 5:**
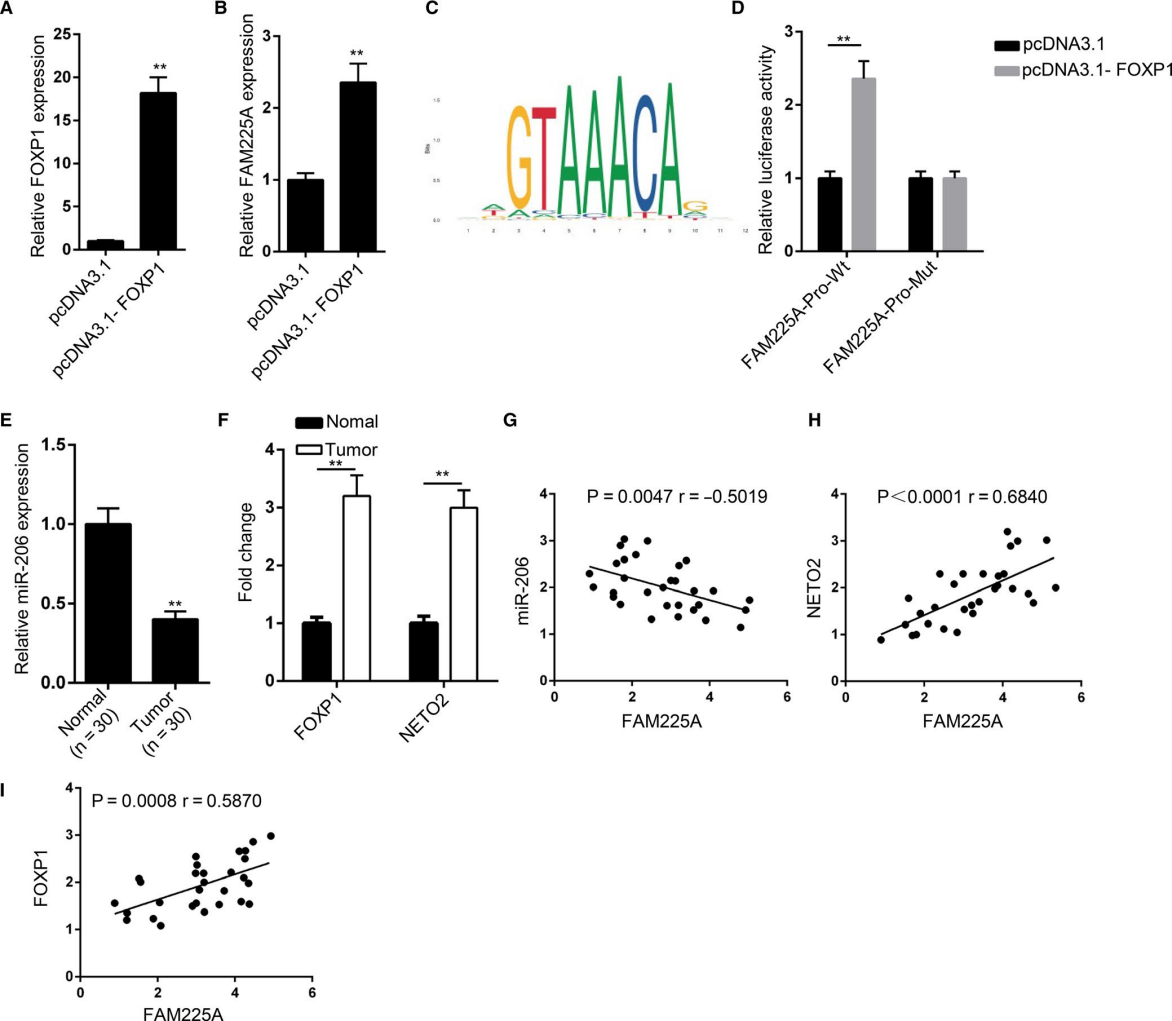
FOXP1 induced FAM225A expression to promote ESCC progression. A, RT‐qPCR was carried out to verify the overexpression efficiency of pcDNA3.1‐FOXP1 in ECA109 cells. B, RT‐qPCR was used to measure the expression of FAM225A after overexpressing FOXP1 in ECA109 cells. C, The binding sequences between FAM225A and FOXP1. D, Luciferase reporter assay was applied to verify the binding ability between FOXP1 and the promoter region of FAM225A. E‐F, The levels of miR‐206, FOXP1, and NETO2 in ESCC tissues were detected by RT‐qPCR assay. G‐I, The correlation between FAM225A and miR‐206 (FOXP1 or NETO2) was assessed by Spearman's correlation analysis. ^**^ *P* < .01

FOXP1 has been reported to be a transcription factor that participates in the regulation of multiple cancers. The overexpression efficiency of FOXP1 was verified (Figure 5A). The upregulation of FOXP1 increased the expression of FAM225A (Figure 5B). The binding sequences between FAM225A and FOXP1 are shown in Figure 5C. The luciferase reporter assay revealed that upregulated expression of FOXP1 notably enhanced the luciferase activities of pGL3‐FAM225A‐WT vectors, while there was no effect on the luciferase activities of pGL3‐FAM225A‐Mut vectors (Figure 5D). Additionally, it was confirmed that miR‐206 was downregulated, and the expression of FOXP1 and NETO2 were upregulated in ESCC tissues (Figure 5E,F). Furthermore, we verified that FAM225A was negatively correlated with miR‐206, and FAM225A was positively correlated with NETO2 (or FOXP1) (Figure 5G‐I). These data suggested that FOXP1 induced FAM225A expression in ESCC.

### 3.6 FAM225A accelerated ESCC progression by regulating NETO2 and FOXP1 expression

**FIGURE 6:**
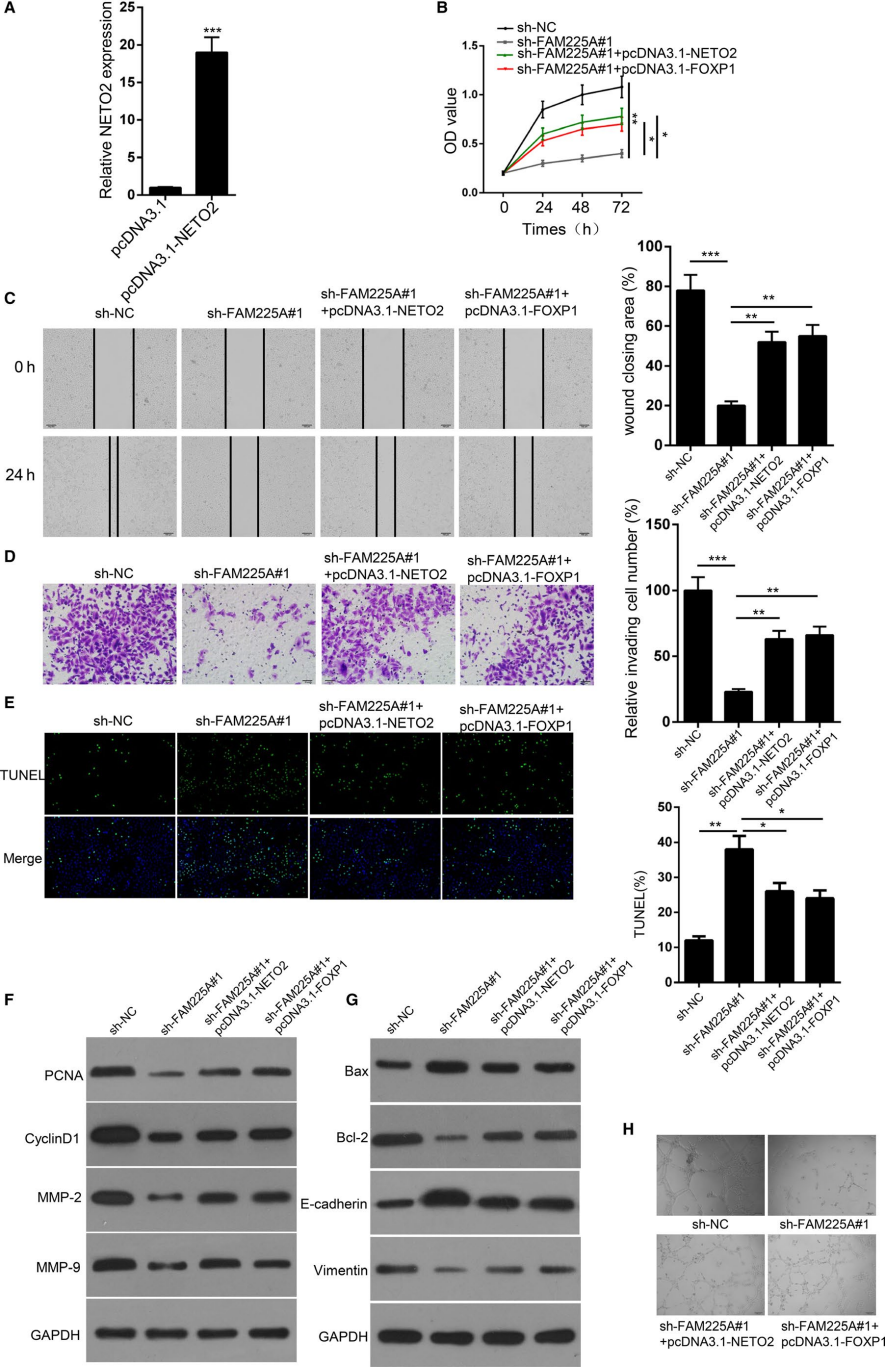
FAM225A accelerated ESCC progression by regulating NETO2 and FOXP1 expression. A, RT‐qPCR assay was used to determine the overexpression efficiency of pcDNA3.1‐NETO2 in ECA109 cells. B, CCK8 assays were used to measure cell proliferation in ECA109 cells transfected with sh‐NC, sh‐FAM225A#1, sh‐FAM225A#1 + pcDNA3.1‐NETO2 or sh‐FAM225A#1 + pcDNA3.1‐FOXP1. C‐D, Wound healing and Transwell assay were performed to assess cell migration and invasion in ECA109 cells transfected with sh‐NC, sh‐FAM225A#1, sh‐FAM225A#1 + pcDNA3.1‐NETO2 or sh‐FAM225A#1 + pcDNA3.1‐FOXP1. E, TUNEL assay was conducted to verify cell apoptosis in ECA109 cells transfected with sh‐NC, sh‐FAM225A#1, sh‐FAM225A#1 + pcDNA3.1‐NETO2 or sh‐FAM225A#1 + pcDNA3.1‐FOXP1. F‐G, Western blot assay was devoted to test the expression of PCNA, CyclinD1, MMP‐2, MMP‐9, Bax, Bcl‐2, E‐cadherin, and Vimentin in ECA109 cells transfected with sh‐NC, sh‐FAM225A#1, sh‐FAM225A#1 + pcDNA3.1‐NETO2 or sh‐FAM225A#1 + pcDNA3.1‐FOXP1. GAPDH serves as a loading control. H, Tube formation assay was devoted to determine the tube formation capacity of HUVECs in sh‐NC, sh‐FAM225A#1, sh‐FAM225A#1 + pcDNA3.1‐NETO2 or sh‐FAM225A#1 + pcDNA3.1‐FOXP1 group. *P < .05, **P < .01, ***P < .001

Next, we conducted rescue assays to confirm whether FAM225A accelerated ESCC progression by regulating NETO2 and FOXP1 expression. NETO2 expression was remarkably increased in ECA109 cells transfected with pcDNA3.1/NETO2 (Figure 6A). Overexpression of NETO2 or FOXP1 abrogated the inhibitory effect of FAM225A silence on cell viability (Figure 6B). FAM225A downregulation notably retarded cell migration and invasion, which was restored by overexpressing NETO2 or FOXP1 (Figure 6C,D). Suppression of FAM225A significantly enhanced cell apoptosis, which was abolished by NETO2 or FOXP1 overexpression (Figure 6E). Additionally, upregulation of NETO2 or FOXP1 could save the effects of FAM225A knockdown on PCNA, CyclinD1, MMP‐2, MMP‐9, Bax, Bcl‐2, E‐cadherin, and Vimentin expression (Figure 6F and G). Additionally, the upregulation of NETO2 or FOXP1 could rescue the inhibitory role of FAM225A knockdown on tube formation (Figure 6H). Overall, the obtained data demonstrated that FAM225A accelerated ESCC progression and angiogenesis by regulating NETO2 and FOXP1 expression.

### 3.7 High level of FAM225A resulted in the poor prognosis of ESCC patients

**FIGURE 7:**
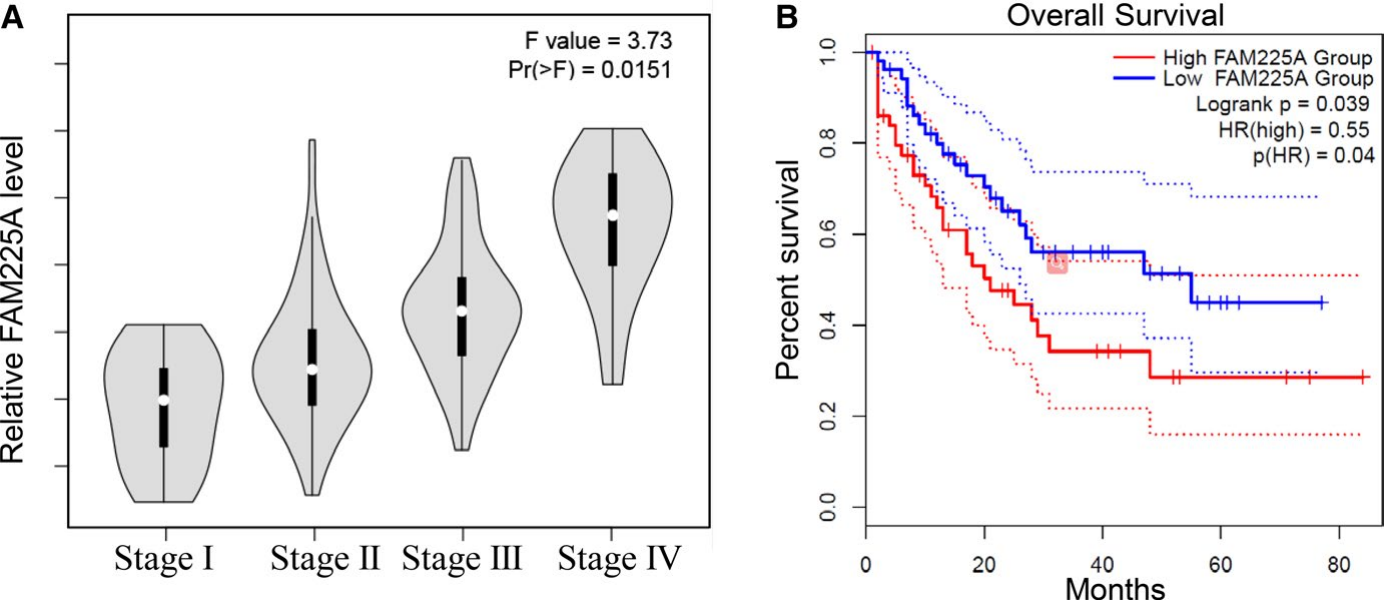
High level of FAM225A resulted in the poor prognosis of ESCC patients. A, The expression of FAM225A in the TNM stage of ESCC patients was measured. B, The survival rate of ESCC patients with high or low level of FAM225A was evaluated

In the end, we further investigated the clinical value of FAM225A in ESCC. Our findings revealed that FAM225A exhibited higher expression in advanced stages (Figure 7A). Furthermore, ESCC patients with a high level of FAM225A had a short survival time (Figure 7B). These findings indicated that the upregulation of FAM225A resulted in the poor prognosis of ESCC patients.

## 4 DISCUSSION

Over the last decade, lncRNAs have attracted a lot of attention and displayed their vital roles in a variety of cancers, especially in ESCC. For instance, lncRNA ATB targets the miR‐200b/Kindlin‐2 axis to facilitate the malignancy of ESCC.^18^ LncRNA TUG1 enhances cisplatin resistance by regulating Nrf2 in ESCC.^19^ LncRNA LINC00675 represses ESCC development and EMT via blocking Wnt/β‐catenin signaling.^20^ FAM225A is a new lncRNA and has only been explored in nasopharyngeal carcinoma to accelerate tumorigenesis.^10^ However, the FAM225A‐mediated regulatory mechanism in ESCC is still unknown. In our work, it was discovered that FAM225A exhibited higher expression in ESCC tissues and cells. The silence of FAM225A attenuated cell viability, migration, and invasion, and promoted cell apoptosis.

LncRNAs have been revealed to be involved in tumor angiogenesis, which is an important link to maintain tumorigenesis and accelerate metastasis.^21^, ^22^, ^23^ Exosomes mediate cellular communications in cancers by transmitting active molecules, including lncRNAs. In lung cancer, lower expression of lncRNA GAS5 in the exosomes promotes tumor angiogenesis.^24^ Exosomal MALAT1 predicts poor prognosis and accelerates angiogenesis in epithelial ovarian cancer.^25^ Glioma cells release lncRNA CCAT2‐contained exosomes to facilitate angiogenesis and restrain endothelial cell apoptosis.^26^ In this work, it was found that exosome‐mediated transfer of lncRNA FAM225A was found in ESCC.

Competing endogenous RNA (ceRNA) network exhibits its regulatory function in human cancers.^27^, ^28^ For instance, lncRNA RP4 absorbs miR‐7‐5p by serving as a ceRNA in colorectal cancer.^29^ LncRNA AFAP1‐AS1 sponges miR‐423‐5p to accelerate nasopharyngeal carcinoma metastasis by acting as a ceRNA.^30^ The lncRNA MIR210HG sponges miR‐1226‐3p to increase mucin‐1c expression thus facilitates tumor metastasis in breast cancer.^31^ Previous researches have demonstrated that miR‐206 is relevant to cancer regulation, including ovarian cancer, prostate cancer, breast cancer, and cervical cancer.^15^, ^16^, ^17^, ^32^ Consistent with these researches, miR‐206 was verified to have the ability to bind with FAM225A in ESCC.

Moreover, we further discovered that FAM225A absorbed miR‐206 to upregulate NETO2 and FOXP1 expression in ESCC. NETO2 drives metastasis in gastric cancer by activating PI3K/Akt/NF‐κB/Snail axis.^33^ The upregulation of NETO2 promotes colorectal carcinoma progression and predicts poor prognosis.^34^ Reduction of NETO2 expression prevents metastasis and induces apoptosis in human nasopharyngeal carcinoma progression.^35^ Additionally, FOXP1 has been discovered to play a carcinogenic role in tumors. FOXP1 drives ovarian cancer stem cell‐like characteristics by functioning as an oncogene.^36^ Cytoplasmic FOXP1 predicts a poor outcome and is associated with ER and calpain II expression in breast cancer.^37^ Also, FOXP1 also could serve as a transcription factor to activate genes transcription. FOXP1 regulates cell viability in breast cancer by acting as an estrogen‐inducible transcription factor.^38^ We discovered that FOXP1 was not only a downstream target gene of miR‐206, but also induced FAM225A expression by serving as a transcription factor. Eventually, it was revealed that overexpression of NETO2 or FOXP1 rescued the effects of FAM225A repression on ESCC progression and angiogenesis. Moreover, the upregulation of FAM225A predicted poor prognosis in ESCC patients.

**FIGURE 8:**
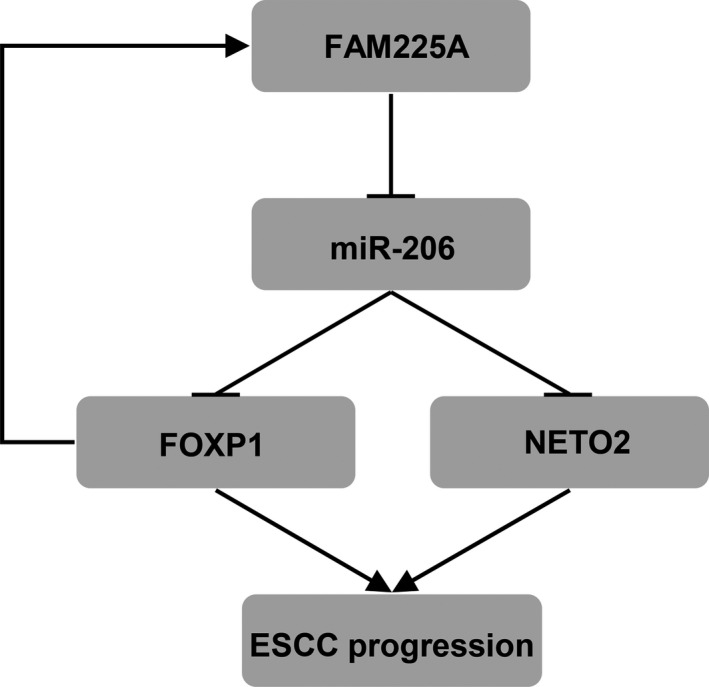
Schematic diagram shows regulatory mechanisms of FAM225A‐induced tumorigenesis of ESCC

To sum up, our results uncovered that FAM225A upregulated NETO2 and FOXP1 expression by sponging miR‐206 and accelerated ESCC progression and angiogenesis (Figure 8). The pleiotropic effects of FAM225A on the pathogenesis of ESCC suggest that it may be a useful therapeutic target for ESCC patients.

## CONFLICT OF INTERESTS

The authors declare that they have no conflict of interests.

## AUTHORS’ CONTRIBUTIONS

Chunyu Zhang, Zhiwei Miao, and Guoqing Shao designed the study. Yan Luo, Jingjing Cao, and Xiaoyu Wang performed the experiments. Chunyu Zhang, Yan Luo, and Jingjing Cao analyzed the data and prepared the figures. Chunyu Zhang, Yan Luo, Zhiwei Miao, and Guoqing Shao drafted the manuscript. All authors approved this manuscript.

## Data Availability

The datasets used and/or analyzed during the present study are available from the corresponding author on reasonable request.
